# Grasping state estimation of printable soft gripper using electro-conductive yarn

**DOI:** 10.1186/s40638-017-0072-4

**Published:** 2017-11-09

**Authors:** Takahiro Matsuno, Zhongkui Wang, Shinichi Hirai

**Affiliations:** Department of Robotics, Ritsumeikan University, Noji Higashi, 1-1-1, Kusatsu, Shiga 525-8577 Japan

**Keywords:** Soft gripper, Estimation, Electro-conductive yarn

## Abstract

Automatic handling of many types of food materials are required to realize the automation of production of commercially prepared box lunches. A printable soft gripper was developed for food handling which is simple to produce with a 3D printer. However, the sensing ability of the printable soft gripper was not discussed in previous research. In this paper, a novel method for estimating the grasping state of a printable soft gripper using electro-conductive yarn is presented. Electro-conductive yarn is a conductive material, and the resistance of strings is changed by stretching. It is less expensive than other sensors that can be used for measurement of grasping state. Additionally, it is easy to assemble and disassemble by hand. Electro-conductive yarn is applied to a prototype printable soft gripper, and the proposed estimation method is verified experimentally. From the experimental results, the estimated grasping state from the resistance of the electro-conductive yarn coincides with the actual grasping state of the gripper. Our proposed method of using electro-conductive yarn was successful for estimating the grasping state of a printable soft gripper.

## Background

Currently, several millions commercially prepared box lunches per day are consumed in Japan [1]. Therefore, automation of the production of the box lunches is required. To produce box lunches, the handling of food using a robot hand must be realized [2, 3]. Hence, a printable soft gripper was developed for food handling which can grasp foods that are soft and easily deformed [4]. It is possible to use a 3D printer to easily produce the soft gripper. For handling of food materials, the estimation of grasping state is necessary. However, the sensing ability of the printable soft gripper to sensing is not discussed sufficiently in previous research [5, 6]. Bending of the printable soft gripper was successfully measured using a strain gage [7]. However, when using strain gages for measuring the bending of the printable soft gripper, the finger size of the gripper is limited by strain gage’s size and the production cost of gripper increases. There are some previous research about pressure sensing, deformation sensing and load sensing for the soft gripper or soft robotics. However, there is no discussion about sensor cost, size and estimation of grasping state [8–10].

In this research, we propose a novel method to estimate the grasping state of the printable soft gripper using electro-conductive yarn. Electro-conductive yarn is a very low cost conductive material, and the resistance of the strings is changed by stretching [11, 12]. Additionally, the sensor size can be determined by users.

In this paper, a concept regarding the estimation of grasping state using electro-conductive yarn is proposed. Then, the calibration method is presented. Finally, the proposed estimation method is applied to prototype printable soft gripper, and estimation result is verified by experiment.

## Estimation method for grasping state of a printable soft gripper

### Concept of a printable soft gripper using electro-conductive yarn

**Fig. 1:**
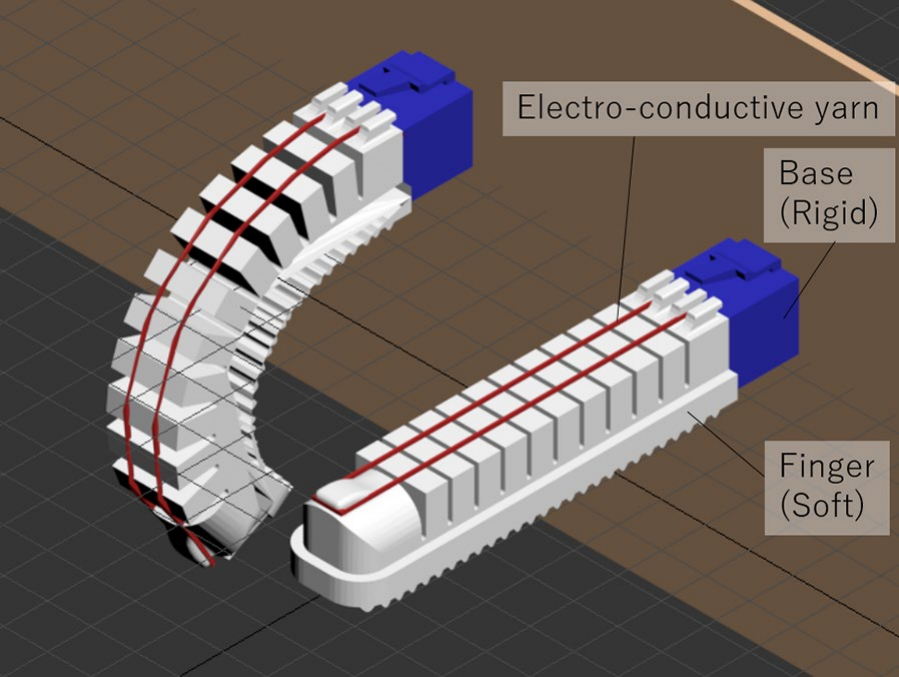
Soft Gripper using electro-conductive yarn

**Fig. 2:**
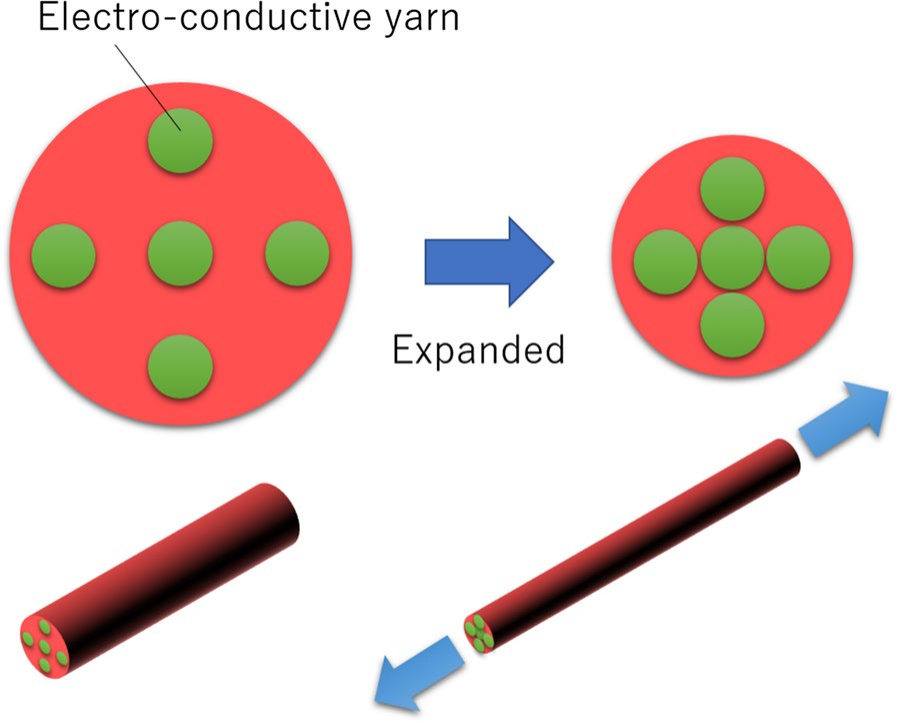
Cross section view of electro-conductive yarn

Our proposed printable soft gripper using electro-conductive yarn and supplementary positioning of electro-conductive yarn is shown in Fig. 1. An electro-conductive yarn is attached to a surface of a finger. When a finger bends, the yarn extends accordingly. Figure 2 shows a cross section view of the electro-conductive yarn. When the air pressure is applied to the printable soft gripper, each chamber expands and the finger is bent. Then, the electro-conductive yarn is extended, and the cross area is decreased by Poisson’s effect. The resistance of the electro-conductive yarn then decreases as each electro-conductive material is contacted, as shown in Fig. 2. The grasping state of the printable soft gripper can be estimated using this change in resistance.

### Approximated model of the printable soft gripper for estimation

**Fig. 3:**
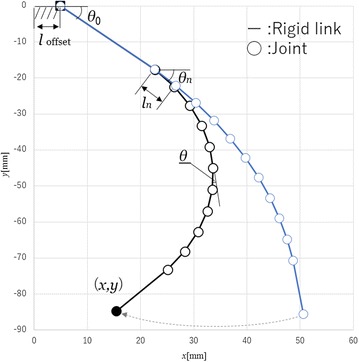
Approximated model of soft gripper

**Fig. 4:**
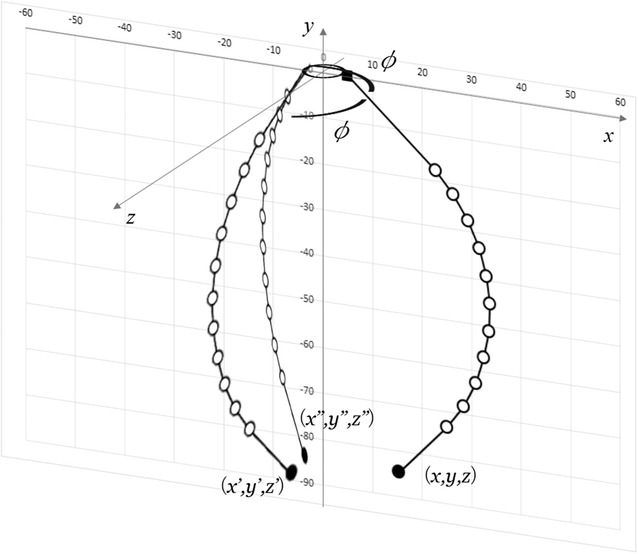
Fingertip positions of each fingers

In this subsection, the geometry of the printable soft gripper is presented. The fingers of soft gripper are approximated as a discrete model as shown in Fig. 3. The chamber section of the soft gripper is approximated as a set of active joints, which are represented by circles. There are few curves that occur in the other section of the soft gripper. Therefore, the gripper can be approximated as a rigid body, which are represented by bars. The tip position of the finger (*x*, *y*) is calculated by:

1 $$\begin{aligned} x= & {} \sum l_n \cos \theta _n + l_{\mathrm {offset}} , \end{aligned}$$

2 $$\begin{aligned} y= & {} \sum l_n \sin \theta _n , \end{aligned}$$

where $l_n, l_{\mathrm {offset}}$ and $\theta _n$ denote the length of the *n*-th link, the length of the offset link and the angle of the *n*-th joint, respectively. For the case of a printable soft gripper, air pressure is applied to individual chambers uniformly. Therefore, each angle bends equally, if there are no other forces applied to the finger. In this paper, *n*-th joint angle $\theta _n$ is assumed as:

3 $$\begin{aligned} \theta _n=n \theta +\theta _0 , \end{aligned}$$

where $\theta$ and $\theta _0$ represents the relative angle of each joints and the absolute angle of first joint, respectively. When using the function of the relationship between the resistances of the electro-conductive yarn and the joint angle, the tip position of the finger (*x*, *y*) is calculated by:

4 $$\begin{aligned} x= & {} \sum l_n \cos (nf(R)+\theta _0) + l_{\mathrm {offset}} , \end{aligned}$$

5 $$\begin{aligned} y= & {} \sum l_n \sin (nf(R)+\theta _0) , \end{aligned}$$

where *R* and $\theta = f(R)$ show the resistance of the electro-conductive yarn and the function of the relationship between the resistance of the electro-conductive yarn and the joint angle, respectively. The gripper has three fingers as shown in Fig. 4. The tip position of finger 2 $(x', y', z')$ and finger 3 $(x'', y'', z'')$ are calculated by:

6 $$\begin{aligned} x'= & {} \left( \sum l_n \cos (nf(R')+\theta _0) + l_{\mathrm {offset}}\right) \cos \phi , \end{aligned}$$

7 $$\begin{aligned} y'= & {} \sum l_n \sin (nf(R')+\theta _0) , \end{aligned}$$

8 $$\begin{aligned} z'= & {} \left( \sum l_n \cos (nf(R')+\theta _0) + l_{\mathrm {offset}}\right) \sin \phi , \end{aligned}$$

9 $$\begin{aligned} x''= & {} \left( \sum l_n \cos (nf(R'')+\theta _0) + l_{\mathrm {offset}}\right) \cos (-\phi ) , \end{aligned}$$

10 $$\begin{aligned} y''= & {} \sum l_n \sin (nf(R'')+\theta _0) , \end{aligned}$$

11 $$\begin{aligned} z''= & {} \left( \sum l_n \cos (nf(R'')+\theta _0) + l_{\mathrm {offset}}\right) \sin (-\phi ) , \end{aligned}$$

where $\phi , R'$ and $R''$ shows the offset angle from finger 1, the resistance of electro-conductive yarn which applied to finger 2 and finger 3, respectively.

### Estimation of approximate diameter of the grasping object

**Fig. 5:**
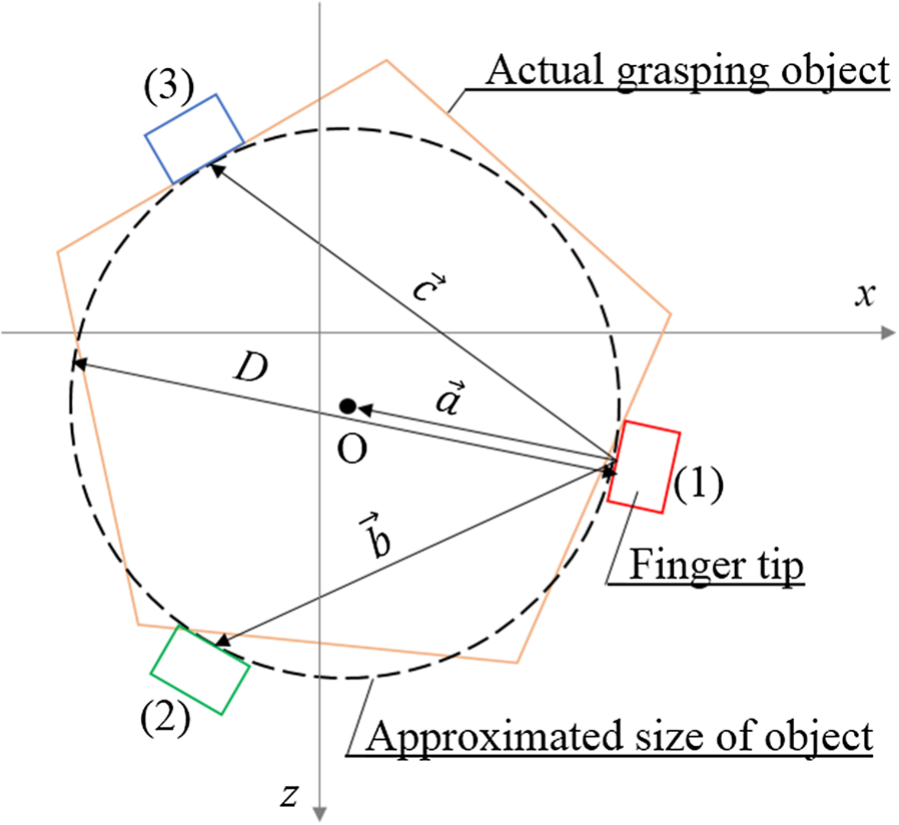
Size estimation of grasping object using each fingertip positions

To estimate grasping state of the gripper, approximate diameter of the grasping object is estimated form each finger’s fingertip position. The approximate diameter *D* is defined as shown in Fig. 5. The vector of fingertip position of finger 1 to center of grasping object $\vec {a}$ is calculated as:

12 $$\begin{aligned} \vec {a}&= u\vec {b} + v\vec {c} , \end{aligned}$$

13 $$\begin{aligned} \vec {b}& = {} \begin{bmatrix} b_x\\ b_z \end{bmatrix} = \begin{bmatrix} x'-x\\ z'-z \end{bmatrix}, \end{aligned}$$

14 $$\begin{aligned} \vec {c}& = {} \begin{bmatrix} c_x\\ c_z \end{bmatrix} = \begin{bmatrix} x''-x\\ z''-z \end{bmatrix}, \end{aligned}$$

where $\vec {b}$ and $\vec {c}$ show the vector of fingertip position of finger 1 to finger 2 and the vector of fingertip position of finger 1 to finger 3, respectively. The variables *u* and *v* are decided by the following two equations:

15 $$\begin{aligned}&(1-2u)\left( b_x^2+b_z^2\right) -2v\left( b_x c_x + b_z c_z\right) = 0 , \end{aligned}$$

16 $$\begin{aligned}&(1-2v)\left( c_x^2+c_z^2\right) -2u\left( b_x c_x + b_z c_z\right) = 0 . \end{aligned}$$

The vector of tip position of finger 1 to center of grasping object $\vec {a}$ is calculated using the values *u* and *v* which fulfill Eqs. (15)–(16). The diameter of grasping object *D* is then calculated as:

17 $$\begin{aligned} D = 2|\vec {a}| . \end{aligned}$$

The grasping state of gripper is estimated by

18 $$\begin{aligned}&{\text {Ready to grasp {:}}}\; D > D_{\mathrm {grasp}} , \end{aligned}$$

19 $$\begin{aligned}&{\text {Successful {:}}}\; D_{\mathrm {grasp}} \ge D > D_{\mathrm {failed}} , \end{aligned}$$

20 $$\begin{aligned}&{\text {Failed {:}}}\; D_{\mathrm {failed}} \ge D , \end{aligned}$$

where $D_{\mathrm {grasp}}$ and $D_{\mathrm {failed}}$ show threshold values for grasping state estimation. When the approximate diameter *D* fulfills Eq. (18), the gripper is opened and it is ready to grasp. When the approximate diameter *D* fulfill Eq. (19), the gripper succeeded to grasp the object. If the approximate diameter *D* fulfills Eq. (20), there are no grasping object. It means that the gripper failed to grasp the object. Using these methods, grasping states of the gripper are estimated.

### Measuring method for resistance of the electro-conductive yarn

**Fig. 6:**
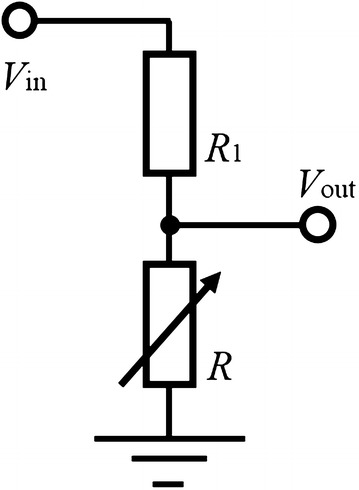
Circuit for measuring

**Fig. 7:**
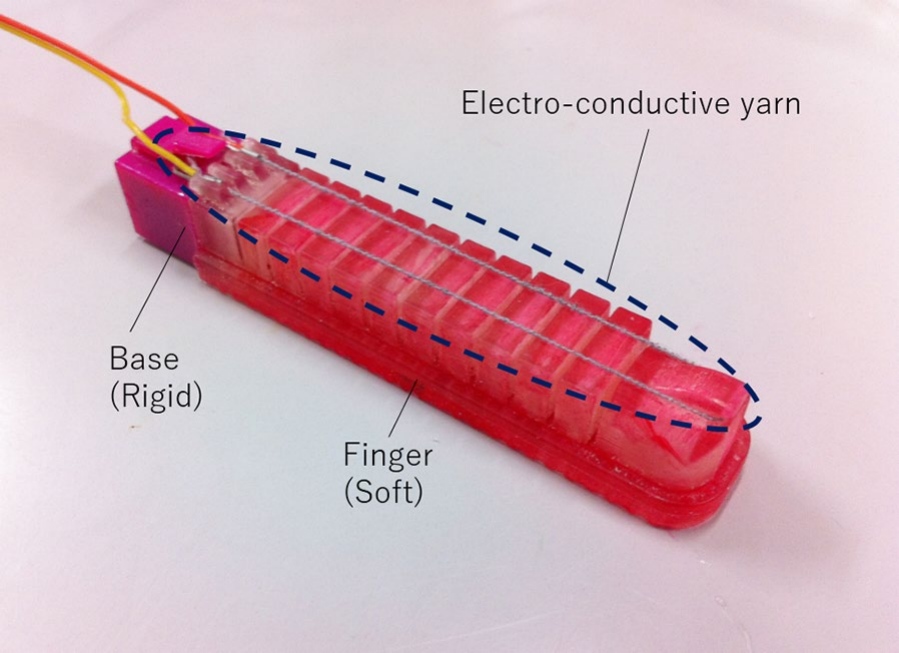
Finger prototype using electro-conductive yarn

The resistance of the electro-conductive yarn *R* is measured in 10 Hz using the circuit which shown in Fig. 6. The output voltage $V_{\mathrm {out}}$ is measured by an AD converter; then, the resistance of the electro-conductive yarn is calculated as:

21 $$\begin{aligned} R=\frac{R_1 V_{\mathrm {out}}}{V_{\mathrm {in}} - V_{\mathrm {out}}} , \end{aligned}$$

where $V_{\mathrm {in}}$ and $R_1$ represent the constant input voltage and constant resistance, respectively. The function describing the relationship between the resistance of the electro-conductive yarn and joint angle $\theta =f(R)$ is calibrated using this method, as discussed in the next section (Fig. 7).

## Calibration method for estimation setup

### Calibration method

In this section, the function of the relationship between the resistance of the electro-conductive yarn and the joint angle is measured experimentally. First, the function of the relationship between the resistance of the electro-conductive yarn and the joint angle is defined by the *m*-th order polynomial function:

22 $$\begin{aligned} \theta = k_mR^m+ \cdots + k_1R+k_0 , \end{aligned}$$

where $k_m$ denotes the coefficients of the polynomial. For the calibration of Eq. (22), several experimental data sets are established. We perform *S* trials with different pressure values applied to the printable soft gripper; then, the resistance of the electro-conductive yarn at *s*-time experiment $R_s$ and the tip position of the finger at *s*-time experiment $(x_s,y_s)$ are measured for each trial. The tip position of the finger from the resistance is estimated by:

23 $$\begin{aligned} x_{\mathrm{{es}}}& = {} \sum l_n \cos \left( n \left( k_mR_s^m+ \cdots + k_1R_s+k_0+\theta _0\right) \right) + l_{\mathrm {offset}} , \end{aligned}$$

24 $$\begin{aligned} y_{\mathrm{{es}}}& = {} \sum l_n \sin \left( n \left( k_mR_s^m+ \cdots + k_1R_s+k_0+\theta _0\right) \right) , \end{aligned}$$

where $(x_{\mathrm{{es}}},y_{\mathrm{{es}}})$ represents the estimated tip position of finger as estimated by the resistance at the *s*-th time experiment. The coefficients of the polynomial $k_m$ are determined by minimizing the least squares of error about $(x_s,y_s)$ and $(x_{\mathrm{{es}}},y_{\mathrm{{es}}})$ as:

25 $$\begin{aligned} {\mathrm {min}}\; \sum \left( (x_s-x_{\mathrm{{es}}})^2+(y_s-y_{\mathrm{{es}}})^2\right) . \end{aligned}$$

Using this method, the function describing the relationship between the resistance of electro-conductive yarn and joint angle can be calibrated.

### Selection of polynomial’s order

In this subsection, the order of the polynomial function for Eq. (22) is selected. If the order of polynomial function is high, Eq. (22) shows a high degree of accuracy, while the time required to estimate the tip position will be longer. Therefore, the order of Eq. (22) should be select a low value. In this study, the tip position of the finger is estimated using several different polynomial orders. Then, the error of the estimated values, necessary time for calibration and necessary time for estimation of grasping state are compared.

**Fig. 8:**
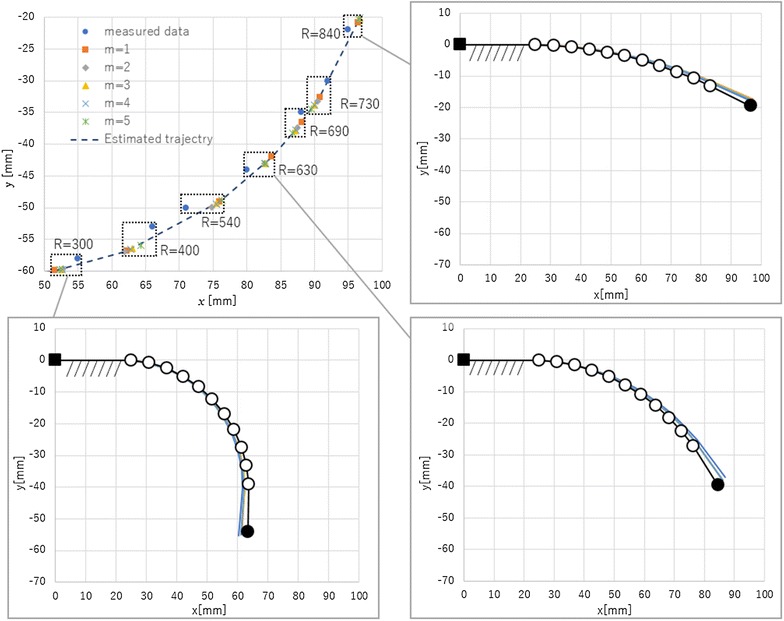
Measured tip position of soft gripper for calibration

The finger of the printable soft gripper using electro-conductive yarn, shown in Fig. 8, was used for the verification of the polynomial order. The electro-conductive yarn is connected to the AD converter of the MCU (STM32F401 Nucleo-64, ST microelectronics, Switzerland) based on the circuit shown in Fig. 6. The resistances of the electro-conductive yarn are calculated by Eq. (21).

**Fig. 9:**
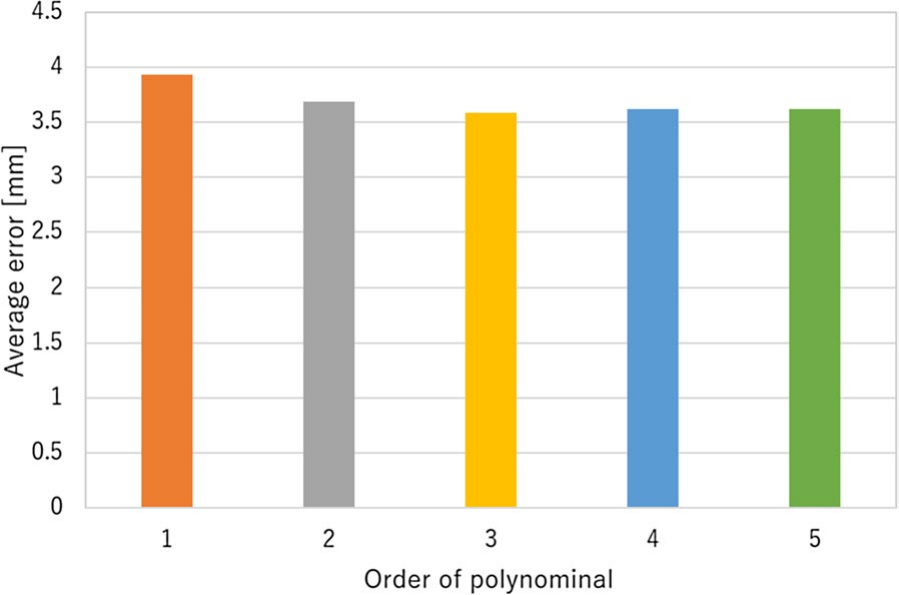
Average error of tip position which are estimated by different order polynomial

**Fig. 10:**
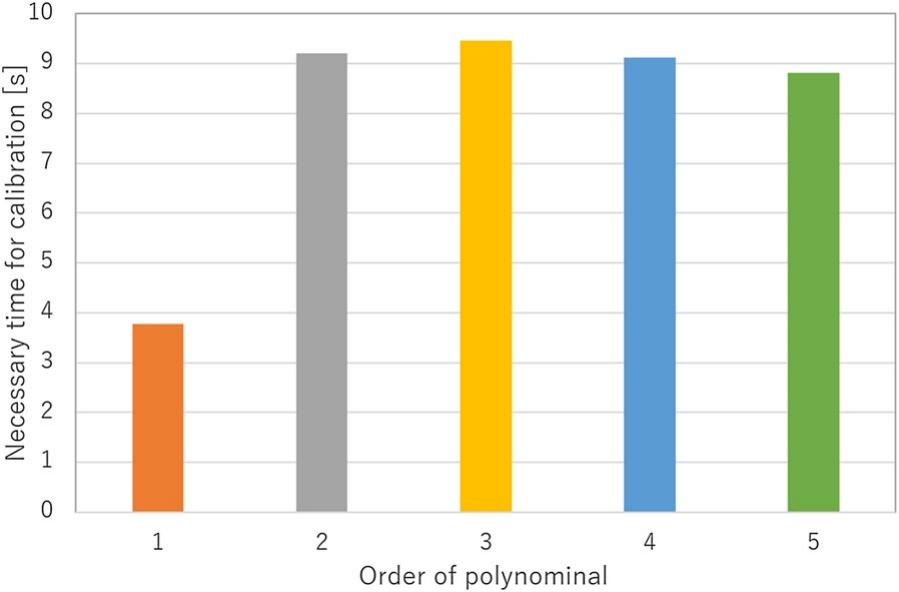
Necessary time for calibration by different order polynomial

**Fig. 11:**
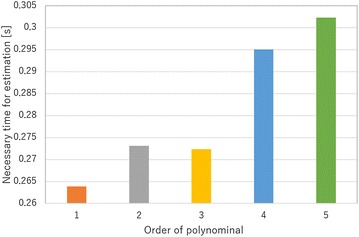
Necessary time for estimation of grasping state by different order polynomial

First, the resistances of the electro-conductive yarn and the tip positions of finger are measured for calibration. In this study, each data point is measured seven times. The measured tip positions of finger are shown in Fig. 8 as blue dots, and the resistances of the electro-conductive yarn in each trial are shown by *R* in Fig. 8. Then, the order values of 1 through 5 are calculated to estimate the tip position. The estimated positions with each polynomial function are shown by the other dots in Fig. 8. Additionally, the average error of the estimated values is shown in Fig. 9. The trajectory of the fingertip is shown in bar line in Fig. 8. The trajectory of fingertip is not affected by the value of polynomial’s order because of the model shown in Eq. (3). The necessary time for calibration is shown in Fig. 10, and the necessary time for estimation of grasping state is shown in Fig. 11. To measure the necessary time for calibration, stopwatch timer function of MATLAB® is used. The resistances of the electro-conductive yarn for calibration are used for measuring necessary time for estimation of grasping state, and each resistance of electro-conductive yarn is assumed to equal value. The estimation is repeated seven times using resistances of the electro-conductive yarn for calibration, and total time for estimation is measured. In this measurement, time for communication is ignored.

From results of Fig. 9, the error of the estimated tip position is not sufficiently decreased, when the order of the polynomial function is increases. From results of Figs. 10 and 11, the time required for estimation will be decreased, when the order of the polynomial function is decreased. Therefore, this paper selected $m = 1$ for Eq. (22).

## Experimental verification

**Fig. 12:**
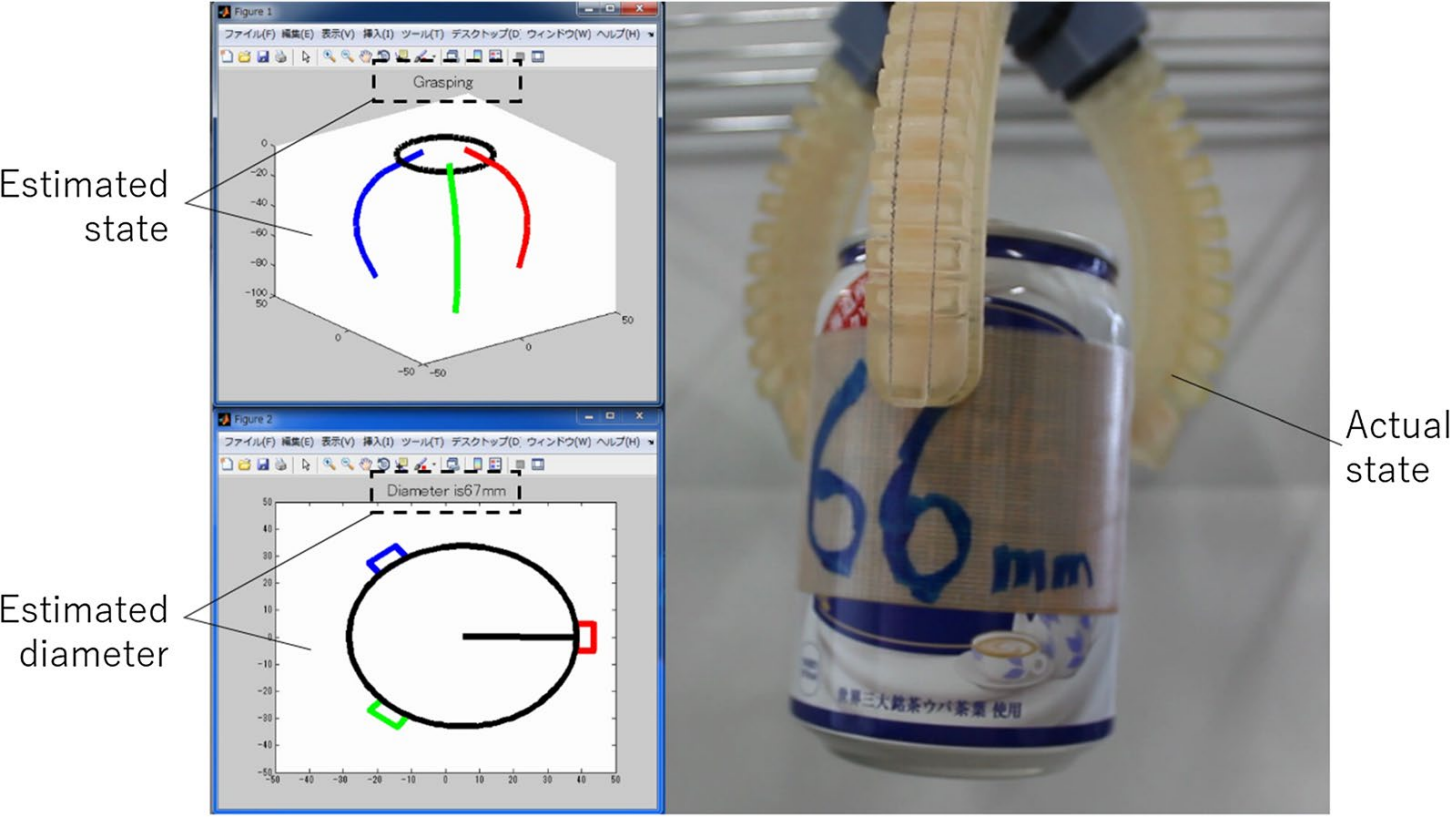
Prototype of soft gripper using electro-conductive yarn

In this section, the electro-conductive yarn is applied to the prototype printable soft gripper. Then, our proposed estimation method is experimentally verified. The prototype of the printable soft gripper using electro-conductive yarn is shown in Fig. 12. The base and fingers of the gripper are printed by a 3D printer (Objet350 Connex3, Stratasys, USA) which can print soft material. The gripper has three fingers, and each finger is fixed to the base at even intervals.

Each finger has electro-conductive yarn, and each resistance is measured by the AD converter of MCU. Additionally, these values are sent to PC where the functions of the relationship between the resistance of each electro-conductive yarn and joint angle are calibrated according to the method discussed in the previous section. The PC estimates the tip position of fingers using Eqs. (4)–(5); then, the estimated shape of fingers and tip position are displayed as Fig. 12. The estimated grasping state and the value of estimated diameter of grasping object are displayed, too. In this research, threshold values for estimation grasping state $D_{\mathrm {grasp}}$ and $D_{\mathrm {failed}}$ are defined as 70 and 30. The measuring sampling time of the resistance is 0.5 s, and approximated diameter of grasping object and tip position of fingers are uploaded in 0.5 s.

**Fig. 13:**
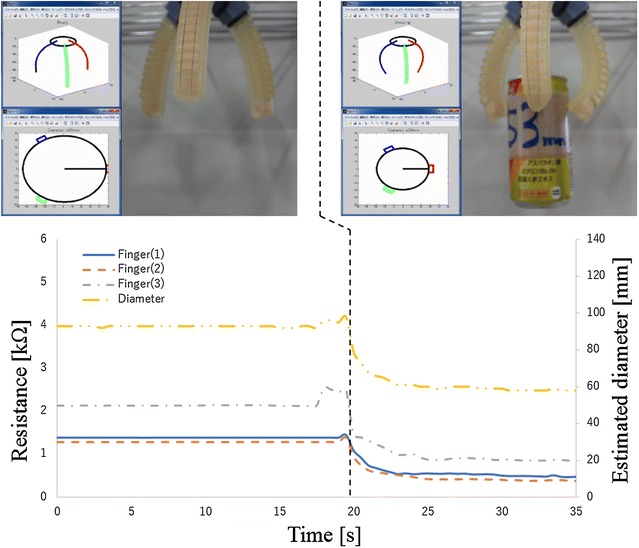
Grasping state estimation using electro-conductive yarn for a cylindrical object

The first experiment verified about the case of succeeded grasping. In this experiment, the cylinder (diameter 53 mm) is used as the target object. The experiment result is shown in Fig. 13. The blue line, red dot line and gray dash-dotted line show the measured resistance values of the electro-conductive yarn. The yellow two-dot chain line shows estimated diameter of grasping object. The estimated diameter is less than 70 mm and larger than 30 mm after 21 s. Therefore, result of estimated grasping state showed that the gripper was grasping the object. It was succeeded to estimate actual grasping state of the gripper.

**Fig. 14:**
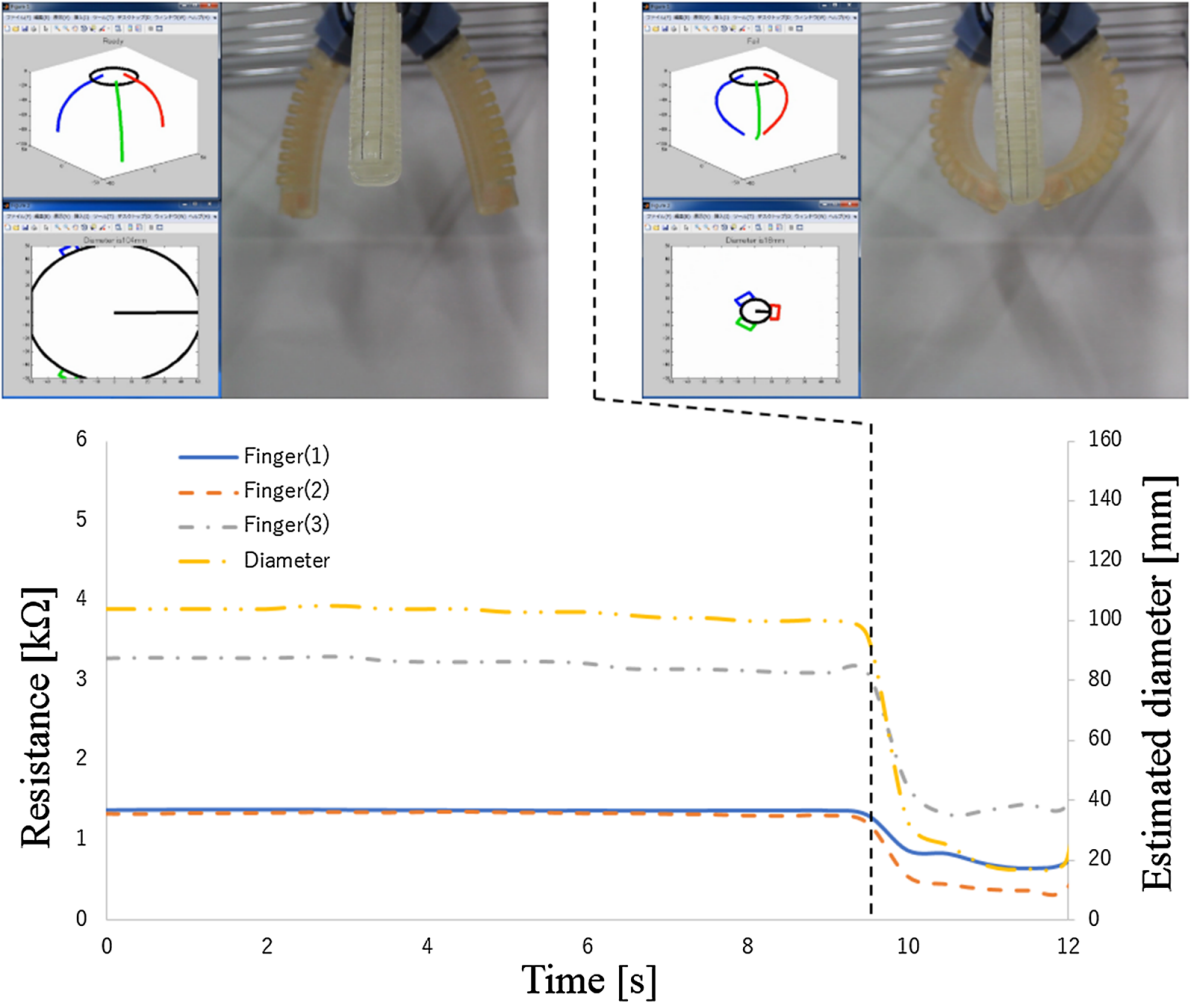
Grasping state estimation using electro-conductive yarn without any object

The second experiment verified about the case of failed grasping. In this experiment, the gripper closes without any object. The experiment result is shown in Fig. 14. The blue line, red dot line and gray dash-dotted line show the measured resistance values of the electro-conductive yarn. The yellow two-dot chain line shows estimated diameter of grasping object. The estimated diameter is less than 30 mm after 11 s. Therefore, result of estimated grasping state showed that the gripper failed to grasp the object. It was succeeded to estimate actual grasping state of the gripper.

**Fig. 15:**
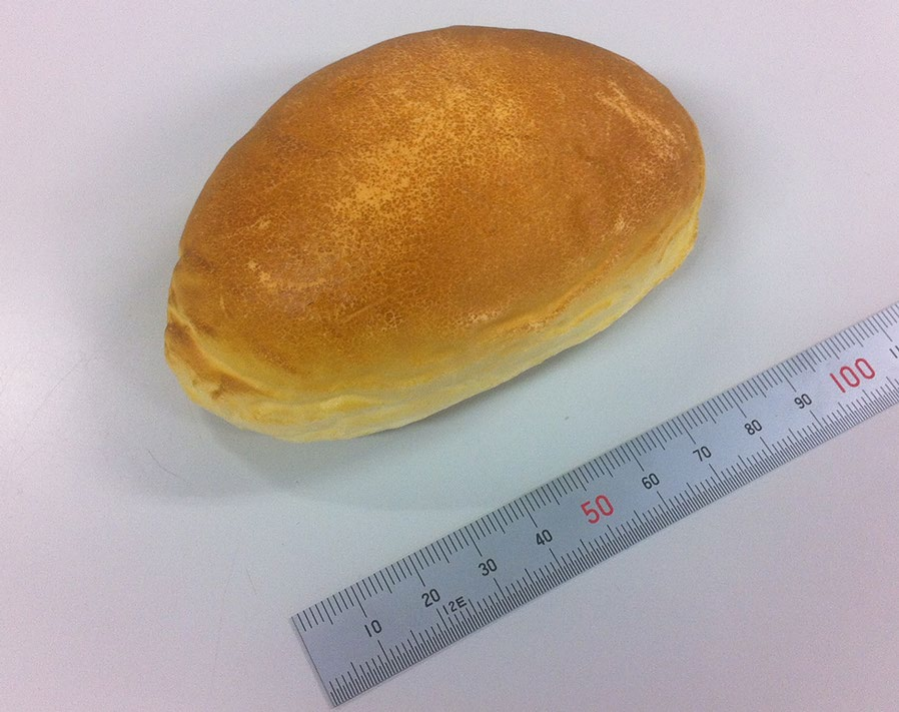
A food sample (bread) for grasping experiment

**Fig. 16:**
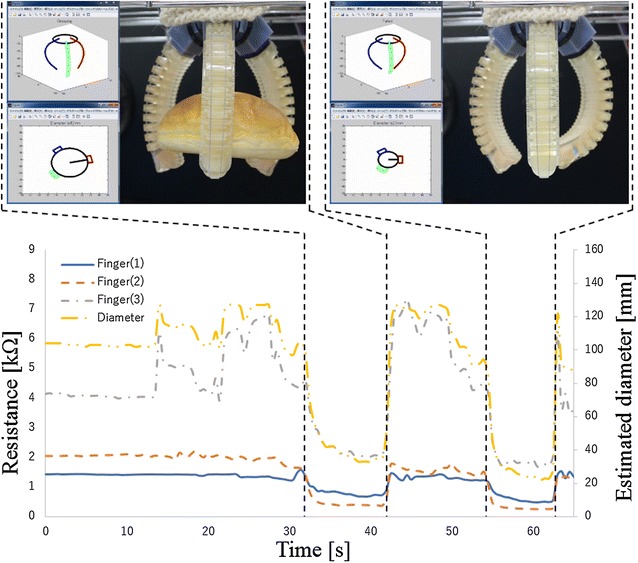
Grasping state estimation using electro-conductive yarn for a bread sample

The final experiment verified about the case of grasping food material. In this experiment, a bread sample as shown in Fig. 15 is used. It is made by soft material of which stiffness is as almost same as real one’s stiffness. In this experiment, the gripper grasps the object at first trial; then, the gripper closed without any object. The experiment result is shown in Fig. 16. The blue line, red dot line and gray dash-dotted line show the measured resistance values of the electro-conductive yarn. The yellow two-dot chain line shows estimated diameter of grasping object. The estimated diameter is less than 70 mm and larger than 30 mm between 31 and 42 s. Therefore, result of estimated grasping state showed that the gripper was grasping the object. After that, the estimated diameter is less than 30 mm between 53 and 61 s. Therefore, result of estimated grasping state showed that the gripper failed to grasp the object. It was succeeded to estimate actual grasping state of the gripper.

From experiment results, the estimated grasping state from the resistance of the electro-conductive yarn is equivalent to the actual gripper condition. Therefore, the proposed method was successful when estimating the grasping state of the printable soft gripper using the electro-conductive yarn. On the other hand, the hysteresis effect of electro-conductive yarn was observed in each experiment. The hysteresis effect of electro-conductive yarn is that the response of resistance changing of yarn is different between stretching and releasing. When the electro-conductive yarn is stretched, the resistance is decrease in leisurely. On the other, when the electro-conductive yarn is stretched, the resistance is increased in quickly. This hysteresis affects time-lag of estimation of grasping state.

## Conclusion

In this paper, a novel method was presented for estimating the grasping state of a printable soft gripper using electro-conductive yarn. Electro-conductive yarn is a conductive material. The resistance of the electro-conductive yarn is changed by stretching the strings. This characteristic is used for estimating the fingertip positions of the printable soft gripper. It is less expensive than other sensors for measuring bending motion. Additionally, it is simple to apply and disassemble a finger. Electro-conductive yarn is applied to top surface of a printable soft gripper. When a printable soft gripper is utilized for grasping, the electro-conductive yarn extends. Then, the resistance of the electro-conductive yarn changes. In our proposed estimation method, the grasping state of the printable soft gripper is estimated from this resistance. The calibration method for the estimation setup is also proposed. Several types of polynomial functions are verified experimentally. Then, the estimation results are compared against the average error. From the comparison results, a linear equation is selected for the proposed calibration method. Finally, the electro-conductive yarn is applied to the prototype of a printable soft gripper, and our proposed estimation method is experimentally verified. From results of the experiment, the estimated grasping states from resistance values of the electro-conductive yarn were validated by the actual conditions of the gripper. Consequently, the proposed method using the electro-conductive yarn was successful for estimating the grasping state of the printable soft gripper. In this study, the estimation time is not optimized and the hysteresis effect is not solved. We will solve these problems, and the estimation speed will be increased in the next step of this research.
